# The mechanical and inflammatory low back pain (MIL) index: development and validation

**DOI:** 10.1186/1471-2474-15-12

**Published:** 2014-01-09

**Authors:** Antonio Cuesta-Vargas, Andre Farasyn, Charles Philip Gabel, Juan V Luciano

**Affiliations:** 1 School Clinical Science at Queensland University of Technology, Brisbane, Australia; 2 Faculty of Health Science, University of Malaga, Malaga, Spain; 3 Department of Human Physiology and Sport Medicine, Vrije Universiteit Brussel (VUB), Brussels, Belgium; 4 Faculty of Science, Health and Education, Centre for Healthy Activities, Sport and Exercise, University of the Sunshine Coast, Sippy Downs, Australia; 5 Parc Sanitari Sant Joan de Déu & Fundació Sant Joan de Déu, Sant Boi de Llobregat, Spain; 6 Red de Investigación en Actividades Preventivas y Promoción de la Salud (RedIAPP), Sant Boi de Llobregat, Spain

**Keywords:** Low back pain, Psychometrics properties, Pain measurement, Screening tool, Inflammatory, Mechanical

## Abstract

**Background:**

The purpose of this study was the development of a valid and reliable “Mechanical and Inflammatory Low Back Pain Index” (MIL) for assessment of non-specific low back pain (NSLBP). This 7-item tool assists practitioners in determining whether symptoms are predominantly mechanical or inflammatory.

**Methods:**

Participants (n = 170, 96 females, age = 38 ± 14 years-old) with NSLP were referred to two Spanish physiotherapy clinics and completed the MIL and the following measures: the Roland Morris Questionnaire (RMQ), SF-12 and “Backache Index” (BAI) physical assessment test. For test-retest reliability, 37 consecutive patients were assessed at baseline and three days later during a non-treatment period. Face and content validity, practical characteristics, factor analysis, internal consistency, discriminant validity and convergent validity were assessed from the full sample.

**Results:**

A total of 27 potential items that had been identified for inclusion were subsequently reduced to 11 by an expert panel. Four items were then removed due to cross-loading under confirmatory factor analysis where a two-factor model yielded a good fit to the data (χ2 = 14.80, df = 13, *p* = 0.37, CFI = 0.98, and RMSEA = 0.029). The internal consistency was moderate (α = 0.68 for MLBP; 0.72 for ILBP), test-retest reliability high (ICC = 0.91; 95%CI = 0.88-0.93) and discriminant validity good for either MLBP (AUC = 0.74) and ILBP (AUC = 0.92). Convergent validity was demonstrated through similar but weak correlations between the ILBP and both the RMQ and BAI (r = 0.34, p < 0.001) and the MLBP and BAI (r = 0.38, p < 0.001).

**Conclusions:**

The MIL is a valid and reliable clinical tool for patients with NSLBP that discriminates between mechanical and inflammatory LBP.

## Background

Low back pain (LBP) is a source of considerable financial and societal costs [1]. Its natural course is argued as either self-limiting, where 3-10% become chronic [2], or recurrent [3] and unfavorable [4], where up to 62% still experience pain after one year [5]. In most cases a specific diagnosis for LBP cannot be defined on the basis of anatomical or physiological abnormalities alone [6]. A subgroup classification approach in RCTs that matches patients with non-specific low back pain (NSLBP) to the treatment they receive, has demonstrated better outcomes than a homogenous classification approach [7]. Consequently, it would seem likely that patients with NSLBP represent a heterogeneous collection of conditions and that subgroup identification with tailored therapies may improve clinical outcomes [8,9]. However, attempts to achieve this through the use of an anatomical or physiological basis have not been demonstrated as being significantly more effective than other approaches [7]. It is crucial to identify subgroups within the broad NSLBP classification on the basis of physical signs and symptoms [10].

Over the last decade there has been a tendency in manual therapy subgroups to conceptualize and manage NSLBP as “mechanical” and/or “inflammatory” [11,12]. Although these labels do not have universally accepted definitions, there is evidence to support both mechanical and inflammatory factors as being involved in the generation of NSLBP [13-16]. There are two distinct notionally contrasted approaches that follow this logical separation: “predominant mechanical” treatments such as exercise [6], traction, mobilization and manipulation [9]; and “predominant anti-inflammatory” treatments such as electromodality approaches [17], non-steroidal anti-inflammatory medications and corticosteroid injections [18]. However, exercise also has an anti-inflammatory effect as evidence indicates a protection against chronic diseases with low-grade inflammation such as diabetes and cardiovascular conditions [19].

In the presence of identifiable anatomical or physiological abnormalities, specific therapies or interventions can be utilized. However with NSLBP only, an empirical approach can be employed [20]. Although some reviews of NSLBP treatments have shown the benefits of physical and pharmacological interventions, these studies concede that the effect sizes are often small and the differences are minimal when additional therapy interventions are included [6,21,22]. This apparent lack of effect may be due in part to the classification of NSLBP as a homogenous condition rather than a heterogeneous collection of undefined but differing conditions, some of which may respond to specific therapeutic interventions [8]. An example of this approach is where patients diagnosed with NSLBP may be identified as either mechanical (MLBP) or inflammatory (ILBP) [23]. It would therefore seem advantageous to attempt to divide LBP sufferers into these groups and that they may respond more readily to separate treatment approaches.

The a*-priori* hypothesis of this study was that a new tool with two dimensions could be developed in order to distinguish between LBP of a Mechanical (MLBP) and inflammatory (ILBP) source. The specific objectives of this study were three-fold: (1) to propose a two-factor model representing MLBP and ILBP levels by exploratory factor analysis (EFA); (2) to ratify this model with confirmatory factor analysis (CFA); and (3) to utilize the CFA results in order to construct and validate summative scales of the standardized values of the index that facilitate assessment of MLBP and/or ILBP.

## Methods

### Design

A two-phase prospective, observational study was conducted involving the development, and subsequent validation of a Mechanical and Inflammatory low back pain (MIL) index.

### Phase 1: Mechanical and Inflammatory LBP (MIL) Index development

A total of 27 items indicating signs and symptoms of potential mechanical and inflammatory NSLBP were extracted from the Walker and Williamson study [23] and assembled in a usable, testable format Additional file 1. A panel with five experts was formed as a part of the content validity assessment and included a sports physician, rheumatologist, general practice physician and two physiotherapists. Each panel member was experienced in treating back pain, had worked in both the clinical and research environments and presented their opinions as a representation of their field of expertise and qualification.

This panel identified areas of omission and item improvement or modification through a consensus approach using the content validity guidelines of a minimum of four votes with an average score of 3 on a four-point ordinal scale. This enabled a diverse and balanced approach that minimized medical or health management bias. This procedure yielded an initial MIL Index with 11 item items.

#### Content validity

A four-point ordinal rating scale was used to rate each of the 11 items: “1” = not relevant, “2” = unable to assess relevance without item revision, “3” = relevant but needs minor alteration, “4” = very relevant and succinct. The item evaluation content validity index [24] calculations were applied to both the items and the entire instrument with an *a-priori* requirement of 3 points with four panel votes.

#### Face validity

A 5-point numerical rating scale was used (0 = not easy, 4 = very easy) to evaluate item accuracy, comprehensiveness and ease of response with an *a-priori* requirement of 3 points.

### Phase 2: Mechanical and inflammatory LBP index (MIL) validation

#### Design

A prospective observational study investigated the responses of participants (n = 170) recruited for the study. Three instruments and one physical test were administered: the Roland-Morris Questionnaire (RMQ), the Short-form Health Status survey (SF-12) and the newly created MIL. The “Backache Index” (BAI) was used as the physical test. The evaluators were two physiotherapists with more than 2-years of professional experience. For test-retest reliability two separate test periods were used on a subgroup of participants (n = 37) with a three-day interval. On each test occasion the second assessment assessor was blinded to the original scores to ensure independent data collection.

#### Patients and setting

The participants (n = 170, 38 ± 14 years-old, n = 96 females) were diagnosed with NSLBP using Waddell’s classification for acute and chronic conditions [20] by a general practitioner (GP), and then were referred to two Spanish physiotherapy outpatient clinics. Exclusion criteria were refusal to participate in the study, LBP as a result of a specific spinal disease, infection, presence of a tumor, osteoporosis, fracture, structural deformity, inflammatory disorder, radicular symptoms or cauda equina syndrome. The study was authorized by the Ethics and Research Committee of the Faculty of Medicine at Malaga University. All participants gave written informed consent, confidentiality and anonymity were preserved at all times, and the principles of the “Declaration of Helsinki” and its subsequent updates were respected.

The standardized measures administered in the study are described below:

1. The Roland-Morris Questionnaire (RMQ) [25] is a 24 item dichotomous scale used to indicate functional disability with a score range from 0 (no disability) to 24 (maximum disability). The cut-offs are determined at 8/24 points for Low to Moderate disability and 16/24 for high disability [26]. The Spanish version has high reliability (ICC = 0.87) [27].

2. The Short-form Health Status survey (SF-12) [28] is a 12-item questionnaire designed to estimate general health status based on physical and mental components (SF-12 PCS and SF-12 MCS). The reliability of the Spanish version is documented with an ICC = 0.90 [28].

3. The Mechanical and Inflammatory LBP Index **(**MIL) was the 11-item draft. The items used in each sub-section are 1) *Mechanical* - pain on trunk flexion, pain on lateral bending and palpation pain (spinous process); 2) *Inflammatory* - intermittent pain during the day, morning pain on waking and initial getting up, stiffness after resting and pain on repetitive bending. Scoring is performed by use of the standardized scores with regression methods determined from factor analysis.

### Physical tests used in the study

The “Backache Index” (BAI) [29] determines the physical status from a single test of 5 simple trunk movements of a patient standing still in erect position: (1) flexion (with knee flexion limited to 10 degrees), (2) bilateral side-flexion to the left and (3) to the right, and (4) bilateral combined extension and lateral flexion to the left and (5) to the right. Observer assessment is performed by means of scoring pain factors obtained by asking the patient, and stiffness estimation at the end of the 5 trunk motions assessed by a physiotherapist according to the BAI criteria [29]. The results are recorded with a four-point score per outcome (0–3 points) and the sum of the five outcomes yields the BAI with a maximum of 15 points. Reliability coefficients of the Spanish version of BAI were excellent (n = 42; ICC = 0.97 at three-day follow-up) [30].

### Statistical analyses

The LISREL v.8.0 and Statistical Package for the Social Sciences (SPSS) v.17.0 were used to compute the statistical analyses. The factor structure, internal consistency, and construct validity were assessed from the full sample. The test-retest reliability was assessed through the Intra-class Correlation Coefficients (ICC) Type 2, 1, and expressed with 95%CI using scores on the MIL from participants at baseline and three days later during a non-treatment period. Participants rating on an 11-point numerical rating scale (NRS) of perceived overall status at baseline and on day three provided the reference criterion to determine change. The subsample of participants (n = 37) for test-retest reliability was determined from the calculations of power analysis from the sample size attributes [31].

The participants were initially randomized into two equal groups for the purpose of cross-sample validation, allowing for exploratory factor analysis (Maximum Likelihood using Oblimin rotation and Kaiser’s normalization) with one half and confirmatory factor analysis with the other.

The “Root Mean Square Error of Approximation” (RMSEA), the “Comparative Fit Index” (CFI), and the “Normed Fit Index” (NFI) are used to evaluate the model fit. For the RMSEA, ≤0.08 reflects a reasonable fit [32]. The NFI and CFI varied along a continuum of 0 to 1 with ≥0.90 being satisfactory [33]. Since components/factors of signs and symptoms of LBP are continuous variables and factor loadings obtained by CFA cannot be used directly to assess the MLBP ILBP factors, a MLBP and ILBP index was developed. This is calculated as the sum of the standardized scores with regression methods of the two factors that comprise our proposed model.

In order to know whether the MIL instrument measures relatively specific constructs, the corrected item-total correlations were examined. Then, the internal consistency of the dimensions was determined by means of Cronbach's α. Test-retest reliability was performed at three days during a period of no treatment [34]. Correlating the BAI, SF-12, RMQ and MIL measures assessed convergent validity. Discriminant validity was determined examining the receiver operating curves (ROC) area under the curve (AUC) values [35].

### Sample size

The minimum *sample sizes* for the validation study were verified from the results as determined from an 80% chance of detecting goodness of fit with an Effect size w = 0.5, alpha = 0.05, beta = 0.08, allowing for 15% attrition. This gave convergent validity (n = 61), test-retest reliability (n = 36), discriminant validity (n = 52) and the pooled samples for internal consistency and factor analysis (n > 100) [31].

### Practical characteristics

Readability was assessed using the Flesch-Kincaid grading scale, a recognised measurement standard that is obtained within the grammar section of most standard word-processing software [36]. Missing responses were determined from all participant responses. Completion and scoring times were determined respectively from participants and clinicians from the average of three separate scores.

## Results

### Phase 1: The MIL development

#### Content validity

The 27 signs and symptoms items were reduced to an initial set of 11 through panel feedback and consensus agreement as detailed (Table 1). The reduction to the final set of seven items was achieved through factor analysis where four items were removed to leave the final MIL 7-item version. Two content validity index calculations were performed on both the items and the complete questionnaire to determine whether an item would be removed due to cross-loading (the presence of an item in both dimensions where loading is > 0.40).

**Table 1:** Set of 11 items obtained through panel feedback and consensus agreement

**Intermitent pain during day**	**Pain on trunk flexion**
Morning pain on waking and initial getting up	Pain on lateral bending
Stiffness after resting	Palpatory pain of vertebrae
Pain on repetive bending	*Pain when standing for a while*
*Palpatory pain of muscles*	*Pain on trunk extension*
*Pain getting out of a chair*	

#### Face validity

All panel members agreed on the MIL being suitably indicative of a questionnaire to determine the presence of mechanical or inflammatory symptoms. All participants were able to complete the MIL without missing responses or additional assistance.

### Phase 2: MIL validation

#### Psychometric characteristics

##### Factor analyses

**Figure 1:**
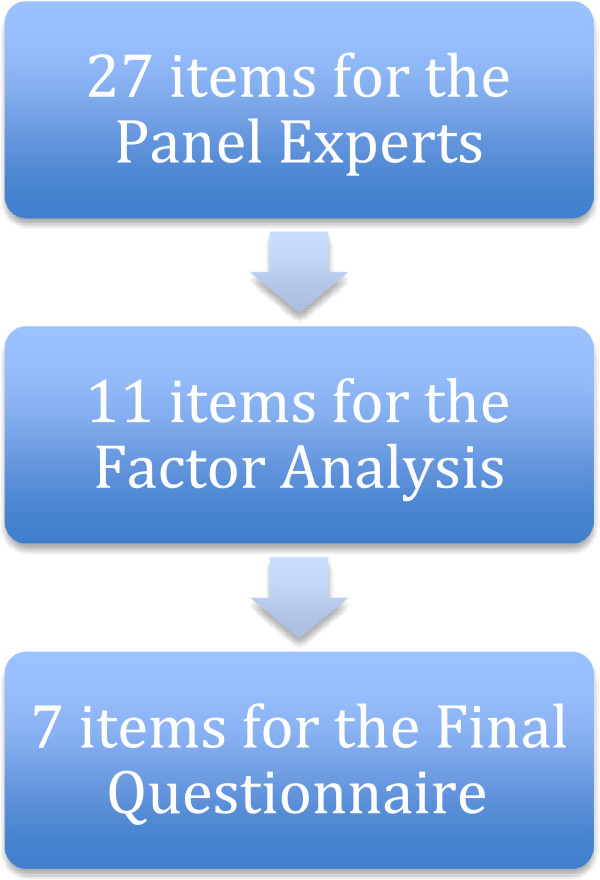
Flow chart of how the final MIL was developed from the initial 27-item version to the final 7-item version.

Four items presented at >0.40 in both dimensions and these items were removed for cross-loading: *“Pain when standing for a while”; “Pain on trunk extension”; “Palpatory pain of muscles”; and “Pain getting out of a chair”.* A flow chart of how the final MIL version was constructed and reduced from the initial 27-items to 7-items is presented (Figure 1).

The Kaiser-Meyer-Olkin measure produced a coefficient of 0.68, indicative of sampling adequacy, and the Bartlett's Test of Sphericity reached statistical significance (p < 0.001). Both supporting the factorability of the correlation matrix. There were ‘two factors’ prior to the ‘inflection’ point in the scree test with Eigenvalues >1.0, item-variance >5% [31], and a total cumulative variance of 51.7%. The rotated ‘two-factor’ solution showed strong loadings (Table 2).

**Table 2:** Structure matrix for the MIL after removing 4 items that presented cross loadings (n = 170)

	**EFA components**	
**MIL items**	**ILBP**	**MLBP**
1. Intermitent pain during day	.61	.26
2. Morning pain on waking and initial getting up	.76	.20
3. Stiffness after resting	.61	.01
4. Pain on repetive bending	.71	.20
5. Pain on trunk flexion	.33	.76
6. Pain on lateral bending	.36	.75
7. Palpatory pain of vertebrae	-.06	.72

Note: Extraction Method = Maximum Likelihood; Rotation Method: Oblimin with Kaiser Normalization.

Items were originally in Spanish and have been translated into English for this manuscript.

ILBP: inflammatory LBP; MLBP: mechanical LBP.

**Figure 2:**
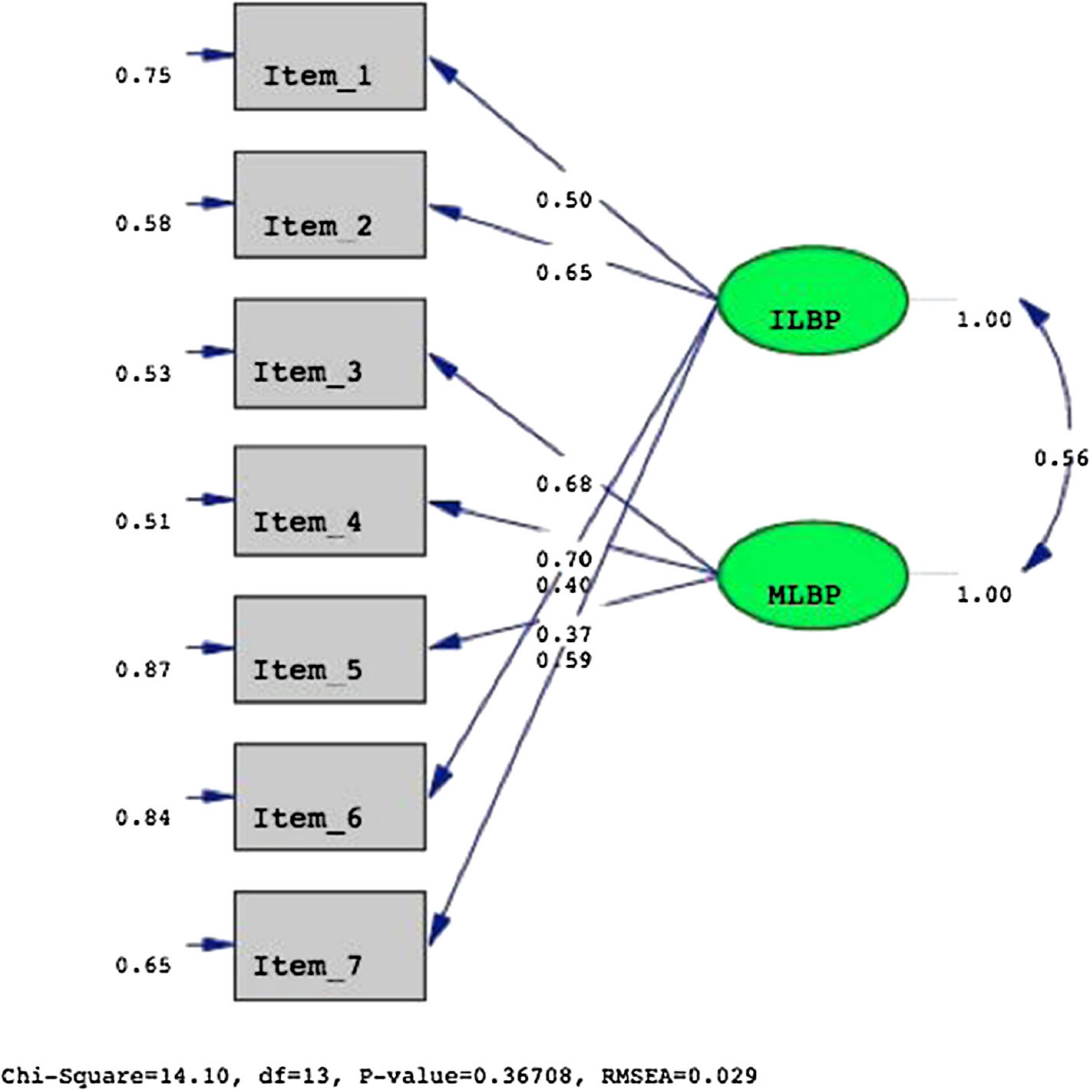
The pathways, factor loading and goodness-of-fit indexes of the two-factor structure underlying the MIL.

The CFA of the two-factor model yielded a non-significant χ^2^-test (χ2 = 14.80, df = 13, *p* = 0.37). The other fit indices were very satisfactory (NFI = 0.97, CFI = 0.98, and RMSEA = 0.029) (Figure 2) and the factor loadings of all variables were >0.40. The correlation coefficient between the two dimensions of 0.56 suggests a moderate relationship.

Correlations between item-total factor Kendall´s Tau are shown in Table 3. The items “morning pain on waking” and “pain on repetitive bending”, both correlate highly with the ILBP component of the MIL questionnaire; while “pain on trunk flexion” and “pain on lateral bending” are factors more related to the MLBP component (Table 3).

**Table 3:** Item-total correlations (n = 170)

		**Item 1**	**Item 2**	**Item 3**	**Item 4**	**Item 5**	**Item 6**	**Item 7**
MLBP		.27^**^	.14	-.21^*^	.09	.55^**^	.67^**^	.29^**^
	Sig. (2-tailed)	.001	.090	.012	.257	.000	.000	.001
ILBP		.46^**^	.63^**^	.46^**^	.60^**^	.34^**^	.36^**^	-.03
	Sig. (2-tailed)	.000	.000	.000	.000	.000	.000	.738

** = p < 0.01.

* = p < 0.05.

ILBP: inflammatory LBP; MLBP: mechanical LBP.

Cronbach's α for the MLBP and ILBP factors was modest, being respectively at 0.68 and 0.72. The development of a combined index is justified given that the two factors are significantly and moderately associated. The MIL index is a pragmatic sum of the standardized scores with regression analysis of the two factors.

### Baseline responses and test–retest reliability

Baseline responses demonstrated normalized distribution for the 7-items. Normality was determined and means and variability of all measures are represented (Table 4). The consistency of the ILBP index, MLBP index and MIL score over time was high (ICC = 0.91; 95%CI =0.88-0.93, ICC = 0.93; 95%CI =0.90-0.96; ICC = 0.89; 95%CI =0.86-0.91, respectively).

**Table 4:** Descriptive statistics of all study measures (n = 170)

**Instruments**	**Minimum**	**Maximum**	**M**	**SD**
Backache index (BAI)	0	13	4.36	3.60
Morning back stiffness (MBS)	0	4	1.55	1.36
Roland Morris Questionaire (RMQ)	0	19	7.19	4.35
ILBP Index	−1.31	1.89	.00	1.00
MLBP Index	−3.95	.84	.00	1.00
ILBP_ + _MLBP	−4.22	2.47	.00	1.56

ILBP: inflammatory LBP; MLBP: mechanical LBP.

### Normal reference values as standardized scores of the mechanical and inflammatory low back pain (MIL) index

The median score for the MLBP Index was 0.504. The 20^th^, 40^th^, 60^th^ and 80^th^ percentiles were −0.377, -0.097, 0.577 and 0.713 respectively. The median score for the ILBP Index was −0.344. The 20^th^, 40^th^, 60^th^ and 80^th^ percentiles were −1.028, -0.443, -0.055 and 1.159 respectively. MIL index are calculated as the sum of the standardised scores (MLBP and ILBP) and the values can be classified in five categories: very low, low, average and high, very high. The median score for MIL Index was 0.129. The 20^th^, 40^th^, 60^th^ and 80^th^ percentiles were −1.21, -0.296, 0.402 and 1.515 respectively.

### Convergent validity

The correlations between the factor ILBP, and RMQ and BAI measurements were practically identical but weak (r = 0.34, p < 0.001). The instruments that correlated weakly with the MLBP were the PCS, RMQ and BAI (r = 0.38, p < 0.001). Taking the factors ILBP and MLBP together, a significant but weak correlation is seen with the BAI and the RMQ, but virtually non-existent with the SF-12 PCS and SF-12 MCS (Table 5) apart from a very weak correlation with the PCS value and the combined MIL score.

**Table 5:** Correlations between MIL factors and other specific and general measures (n = 170)

		**ILBP**	**MLBP**	**RMQ**	**BAI**	**SF-12 PCS**	**SF-12 MCS**
ILBP	*r*		.16^*^	.34^**^	.34^**^	.08	.12
	*p*	.	.023	.000	.000	.307	.127
MLBP	*r*	.16^*^		.16^*^	.38^**^	.17^*^	.36
	*p*	.023	.	.024	.000	.026	.070.
ILBP_ + _MLBP	*r*	.72^**^	.44^**^	.34^**^	.46^**^	.16^*^	.13
	*p*	.000	.000	.000	.000	.045	.097

** = p < 0.01.

* = p < 0.05.

ILBP: inflammatory LBP; MLBP: mechanical LBP; RMQ: Roland Morris Questionnaire.

BAI: Backache Index; SF-12 PCS and SF-12 MCS: SF-12 physical and mental components; SF-12: short Form-12 Health Survey.

### Discriminant validity

The ROC analyses indicated that the AUCs (expressed in 95% confidence interval) for the specific low back pain questionnaires were from 0.74-0.92 for the RMQ and 0.51-0.65 for the BAI. In general, no significances were noted with the exception of the ILBP and the ILBP plus MLBP factors in the case of the RMQ value of state variable at 20%.

### Practical characteristics

*Readability* was acceptable with a Flesch-Kincaid grade level at 6.8 and 68.5% reading ease.

*Missing responses* were acceptable with four responses found in three questions (1, 2, and 4) at a frequency of 5%. *Completion time* was 6.57 ± 3.03 minutes.

## Discussion

The findings of this study indicated that the MIL had high reliability and the ability to adequately discriminate patients into two subgroups of MLBP and/or ILBP.

The MLBP characteristics were ‘Pain trunk flexion’, ‘Pain lateral bending’ and ‘Palpation pain of vertebrae’. The ILBP characteristics were ‘Morning pain on walking and initial getting up’, ‘Pain repetitive bending’, ‘Intermittent pain during day’ and ‘Stiffness after resting’.

Provocative symptoms from MLBP elicited by lateral bending may stem from either inflammation of thoraco-lumbar spine articulations, such as disco-vertebral and facet joints, and/or from muscle strain. For ILBP, initiating movements may stress inflamed and swollen soft tissues as well as the local lumbar and sacro-iliac joints, even if no radiological anatomic spine or pelvic abnormalities are evident [37].

Walker and Williamson [22] in their study of NSLBP patients found morning pain on activity suggested high levels of agreement as an indicator of ILBP, while pain when lifting suggested rather MLBP. In this study the ILBP corresponded to “morning pain on waking”, while for MLBP the two elements of trunk “pain on lateral bending” and “flexion” corresponded with BAI. Consequently the combination of these two aspects of mechanical and inflammatory indicators in the MIL index should be able distinguish between ILBP and MLBP and confirm the approach of Walker et al. [22]. This supports the concept of differentiation of NSLBP into these two subgroups.

No strong correlation was found between ILBP and the SF-12 factors (PCS and MCS measures), only a weak correlation with the combined MIL components and that of the PCS score. This confirms the findings of a previous study of Moix et al. [38], where very weak associations were found between chronic LBP and mental health status [38]. The predictive ability of the MIL questionnaire for functional disability was moderate to high.

A pilot study of Riskman et al. [39], that employed the mechanical and inflammatory LBP analogue instrument was unable to effectively categorize the majority of patients into ILBP or MLBP. The MIL by contrast has employed a method that appears more effective at discriminating between these aspects. This may help the clinical decision process regarding the type of loading treatment (pharmacological or mechanical) that would be more effective for patients when the symptom profile is taken into account. This should increase the adequacy of treatment interventions provided to patients.

### Weaknesses and strengths

It is acknowledged that the difference between acute and chronic NSLBP is probably responsible for the weak responsiveness results in the convergent validity. The mix of patients with acute NSLBP represents a bias towards patients with flexion problems while chronic NSLBP represents a bias towards general stiffness [40]. These factors may have increased the variability of the results. The selection of symptomatic items was developed based on the opinions of the panelists and not assessed through an experimental investigation. The strength of this study is that it supports the reliability of the new MIL questionnaire system and the ability to distinguish between ILBP from MLBP subgroups of NSLBP patients.

### Implications and future directions

Our results suggest that the MIL can pragmatically distinguish NSLBP into subgroups of mechanical and inflammatory symptoms. This is achieved through a continuous index based on the components of a two-factor model obtained through CFA. The MIL should be able to offer a standard clinical frame of reference. Furthermore, in order to help clinicians obtain immediate results based on raw patient data, we have developed a software application to provide the index values (see http://www.salud.uma.es/calculaMIL/).

Our study may lead to improvements in the understanding and assessment of mechanical and inflammatory NSLBP. It confirms a two-factor model underlies NSLBP and that clinicians can use a simple index to distinguish between these two subgroups. Further research is needed to determine the generalizability and cross-cultural validity of the MIL. It has potential utility in patient assessment and treatment evaluation as well as the ability to provide clinicians with a quick assessment to distinguish between mechanical and inflammatory NSLBP components. Such research may assist in the demonstration of the value of this new MIL procedure in the clinical setting.

## Conclusions

The findings of this study suggest the MIL, in this initial stage of research, is a valid and reliable for distinguishing between mechanical and inflammatory LBP. While earlier similar studies could not retrieve the difference between mechanical versus inflammatory LBP, the new elaborated MIL scale gives clinicians the opportunity to decide in which direction treatment options should be considered. The main shortcoming in this study in that both acute and chronic NSLBP patients’ were included. Consequently, further studies are needed to assess the generalizability and cross-cultural validity of our findings.

## Abbreviations

ACSM: American College of Sports Medicine; AUC: Area under the curve; BAI: Backache Index; CFA: Confirmatory factor analysis; CFI: The Comparative Fit Index; CLBP: Chronic low back pain; GP: General practitioners; ICC: Intraclass correlation coefficients; ILBP: Inflammatory LBP; LBP: Low back pain; MIL: Mechanical and Inflammatory Low Back Pain; MLBP: Mechanical LBP; NFI: The Normed Fit Index; NRS: Numerical rating scale; NSLBP: Non-specific low back pain; RMSEA: Root Mean Square Error of Approximation; ROC: Receiver operating curves; RMQ: Roland Morris Questionnaire; SF-12 PCS and SF-12 MCS: SF-12 physical and mental components; SF-12: Short Form-12 Health Survey; SPSS: Statistical Package for the Social Sciences.

## Competing interests

The authors declare that they have no competing interests.

## Authors' contributions

AIC-V participated in the design of the study and performed the statistical analysis and to drafted the manuscript. AF, CPG and JVL collected the data and helped to draft the manuscript. All authors read and approved the final version of the manuscript.

## Pre-publication history

The pre-publication history for this paper can be accessed here:

http://www.biomedcentral.com/1471-2474/15/12/prepub

## Supplementary Material

Additional file 1 Initial set of 27 by the panel through removal of duplicates and redundancies. Click here for file

## References

[B1] Ijzelenberg WAB Risk factors for musculoskeletal symptoms and ensuing health care use and sick leave Spine 2005 30 1550 1556 10.1097/01.brs.0000167533.83154.28 15990672

[B2] Carey TS Garrett JM Jackman AM Examination of an Inception Cohort of Patients With Chronic Low Back Pain Spine 2000 25 115 120 10.1097/00007632-200001010-00019 10647169

[B3] Stanton TR How do we define the condition ‘recurrent low back pain’? A systematic review Eur Spine J 2010 19 533 539 10.1007/s00586-009-1214-3 19921522 PMC2899839

[B4] Hestbaek L Leboeuf Y De C Manniche C Low back pain: what is the long-term course? A review of studies of general patient populations Eur Spine J 2003 12 149 165 12709853 10.1007/s00586-002-0508-5 PMC3784852

[B5] Henschke N Prognosis in patients with recent onset low back pain in Australian primary care: inception cohort study BMJ 2008 337 171 10.1136/bmj.a171 PMC2483884 18614473

[B6] Airaksinen O COST B13 Working Group on Guidelines for Chronic Low Back Pain. Chapter 4. European guidelines for the management of chronic nonspecific low back pain Eur Spine J 2006 15 Suppl 2 192 300 10.1007/s00586-006-1072-1 PMC3454542 16550448

[B7] Fersum KV Integration of subclassification strategies in randomised controlled clinical trials evaluating manual therapy treatment and exercise therapy for non-specific chronic low back pain: a systematic review Br J Sports Med 2010 44 14 1054 1062 10.1136/bjsm.2009.063289 19996331

[B8] Fritz JM Delitto A Erhard RE Comparison of classification-based physical therapy with therapy based on clinical practice guidelines for patients with acute low back pain Spine 2003 28 1363 1372 12838091 10.1097/01.BRS.0000067115.61673.FF

[B9] Childs JD A clinical prediction rule to identify patients with low back pain most likely to benefit from spinal manipulation Ann Intern Med 2004 141 920 928 10.7326/0003-4819-141-12-200412210-00008 15611489

[B10] Fritz JM George S The use of a classification approach to identify subgroups of patients with acute low back pain Spine 2000 25 106 114 10.1097/00007632-200001010-00018 10647168

[B11] Ross JS Non-mechanical inflammatory causes of back pain: current concepts Skeletal Radiol 2006 35 7 485 487 10.1007/s00256-006-0121-5 16752161

[B12] DeSanto J Ross JS Spine infection/inflammation Radiol Clin North Am 2011 49 1 105 127 10.1016/j.rcl.2010.07.018 21111132

[B13] Chaudhary N Longworth S Sell PJ Management of mechanical low back pain e a survey of beliefs and attitudes in GPs from Leicester and Nottingham Eur J Gen Pract 2004 10 2 71 72 10.3109/13814780409094238 15232529

[B14] Valat JP Factors involved in progression to chronicity of mechanical low back pain Joint Bone Spine 2005 72 3 193 195 10.1016/j.jbspin.2004.07.010 15850987

[B15] Hurri H Karppinen J Discogenic pain Pain 2004 112 225 238 10.1016/j.pain.2004.08.016 15561376

[B16] Igarashi A Inflammatory cytokines released from the facet joint tissue in degenerative lumbar spinal disorders Spine 2004 19 2091 2095 15454697 10.1097/01.brs.0000141265.55411.30

[B17] Bjordal JM Low-level laser therapy in acute pain: a systematic review of possible mechanisms of action and clinical effects in randomized placebo-controlled trials Photomed Laser Surg 2006 24 2 158 168 10.1089/pho.2006.24.158 16706694

[B18] Saal JS The role of inflammation in lumbar pain Spine 1995 20 1821 1827 10.1097/00007632-199508150-00013 7502140

[B19] Petersen AM Pedersen BK The anti-inflamatory effecto of exercise J Appl Physiol 2005 98 1154 1162 10.1152/japplphysiol.00164.2004 15772055

[B20] Koes B An updated overview of clinical guidelines for the management of non-specific low back pain Eur Spine J 2010 19 2075 2094 10.1007/s00586-010-1502-y 20602122 PMC2997201

[B21] Van Tulder M Exercise therapy is a widely used treatment for low back pain Cochrane Database Rev 2000 2 CD 00335

[B22] Assendelft WJJ Spinal manipulative therapy for low-back pain Cochrane Database Syst Rev 2004 CD000447 14973958 10.1002/14651858.CD000447.pub2

[B23] Walker BF Williamson OD Mechanical or inflammatory low back pain. What are the potential signs and symptoms? Man Ther 2009 14 314 320 10.1016/j.math.2008.04.003 18555728

[B24] Lynn MR Determination and quantification of content validity Nurs Res 1986 6 382 385 3640358

[B25] Roland M Morris R A study of the natural history of low-back pain. Part II: development of guidelines for trials of treatment in primary care Spine 1983 8 2 145 150 10.1097/00007632-198303000-00005 6222487

[B26] Stratford PW Sensitivity to change of the Roland-Morris Back Pain Questionnaire: part 1 Phys Ther 1998 78 11 1186 1196 9806623 10.1093/ptj/78.11.1186

[B27] Kovacs FM Validation of the Spanish version of the Roland-Morris questionnaire Spine 2002 27 5 538 542 10.1097/00007632-200203010-00016 11880841

[B28] Vigalut G Interpretation of sf36 and sf12 questionnaires in Spain: physical and mental components Med Clin 2008 130 19 726 735 10.1157/13121076 18570798

[B29] Farasyn A Meeusen R Validity of the new Backache Index (BAI) in patients with low back pain Spine J 2006 6 5 565 571 10.1016/j.spinee.2006.01.021 16934729

[B30] Cuesta-Vergas A Gonzalez-Sanchez M Farasyn A Development of a Spanish version of the "Backache Index" J Back Musculoskelet Rehabil 2010 23 3 105 110 20858939 10.3233/BMR-2010-0256

[B31] Field A Discovering Statistics using SPSS 2005 London: SAGE Publications Ltd

[B32] Schumacher RE Lomax RGA Beginner’s guide to structural equation modelling. Mahwah 1996 NJ: Lawrence Erlbaum

[B33] McDonald RP Marsh HW Choosing a multivariate model: Noncentrality and goodness of fit Psychol Bull 1990 107 247 255

[B34] Vincent W Statistics in kinesiology 1994 USA: Human Kinetic

[B35] Swets JA Measuring the accuracy of diagnostic systems Science 1988 240 1.285 93 10.1126/science.3287615 3287615

[B36] Paasche-Orlow MK Taylor HA Brancati FL Readability Standards for Informed-Consent Forms as Compared with Actual Readability N Engl J Med 2003 348 721 726 10.1056/NEJMsa021212 12594317

[B37] Steer S Low back pain, sacroiliitis, and the relationship with HLA-B27 in Crohn's disease J Rheumatol 2003 30 3 518 522 12610811

[B38] Moix J Catastrophizing, state anxiety, anger, and depressive symptoms do not correlate with disability when variations of trait anxiety are taken into account. A study of chronic low back pain patients treated in Spanish pain units [NCT00360802]. Spanish Back Pain Research Network Pain Med 2011 12 7 1008 1017 10.1111/j.1526-4637.2011.01155.x 21668743

[B39] Riskman JS Williamson OD Walker BF Delineating inflammatory and mechanical subtypes of low back pain: a pilot survey of fifty low back pain patients in a chiropractic setting Chiropr Man Ther 2011 19 5 1 9 10.1186/2045-709X-19-5 PMC3048575 21299867

[B40] Waddell G Objective clinical evaluation of physical impairment in chronic low back pain Spine 1992 17 6 617 628 10.1097/00007632-199206000-00001 1308095

