# Supplementary material for: The mechanical and inflammatory low back pain (MIL) index: development and validation
Source: BMC Musculoskelet Disord. 2014 Jan 9;15:12. doi: 10.1186/1471-2474-15-12 (PMC3893585; doi:10.1186/1471-2474-15-12)
Supplement: Additional file 1 — Initial set of 27 by the panel through removal of duplicates and redundancies. [file 1471-2474-15-12-S1.docx]

Additional file 1: Initial set of 27 by the panel through removal of duplicates and redundancies

Morning pain on waking

Intermittent pain during day

Pain later in the day

Straight leg raising hurts

Pain wakes the person up

Pain on sitting for a while

Pain when standing for a while

Pain when lifting

Pain bending forward a little

Burning pain

Aching pain

Stabbing pain

Constant pain

Pain on trunk flexion

Pain on trunk extensión

Pain on lateral bending

Palpatory pain of muscles

Palpatory pain of spinous process

Stiffness after resting

(includes sitting)

Morning and afternoon pain

Doing a sit up is painful

Driving long distances is painful

Pain on walking more than 50 m

Pain on running

Pain on repetitive bending

Pain getting out of a chair

Pain on cough
